# No impact of strongylid infections on the detection of *Plasmodium* spp. in faeces of western lowland gorillas and eastern chimpanzees

**DOI:** 10.1186/s12936-017-1822-z

**Published:** 2017-04-26

**Authors:** Mwanahamisi I. Mapua, Barbora Pafčo, Jade Burgunder, Ilona Profousová-Pšenková, Angelique Todd, Chie Hashimoto, Moneeb A. Qablan, David Modrý, Klára J. Petrželková

**Affiliations:** 1 Department of Pathology and Parasitology, University of Veterinary and Pharmaceutical Sciences, Palackého tř. 1946/1, 612 42 Brno, Czech Republic; 2 Faculty of Science, Masaryk University, Kotlářská 267/2, 611 37 Brno, Czech Republic; 3 WWF, Dzanga Sangha Protected Areas, BP 1053 Bangui, Central African Republic; 4 Primate Research Institute, Kyoto University, Kanrin, Inuyama, Aichi 484-8506 Japan; 5 Central European Institute for Technology (CEITEC), University of Veterinary and Pharmaceutical Sciences, Palackého tř. 1946/1, 612 42 Brno, Czech Republic; 6 Institute of Parasitology, Biology Centre, Czech Academy of Sciences, Branišovská 1160/31, 370 05 České Budějovice, Czech Republic; 7 Institute of Vertebrate Biology, Czech Academy of Sciences, Květná 8, 603 00 Brno, Czech Republic; 8 Liberec Zoo, Lidové sady 425/1, 460 01 Liberec, Czech Republic

**Keywords:** Co-infection, Faeces, Strongylid, *Necator* spp., *Plasmodium* spp., Malaria, Western lowland gorilla, Eastern chimpanzee

## Abstract

**Background:**

Although a high genetic diversity of *Plasmodium* spp. circulating in great apes has been revealed recently due to non-invasive methods enabling detection in faecal samples, little is known about the actual mechanisms underlying the presence of *Plasmodium* DNA in faeces. Great apes are commonly infected by strongylid nematodes, including hookworms, which cause intestinal bleeding. The impact of strongylid infections on the detection of *Plasmodium* DNA in faeces was assessed in wild, western, lowland gorillas from Dzanga Sangha Protected Areas, Central African Republic and eastern chimpanzees from Kalinzu Forest Reserve, Uganda.

**Methods:**

Fifty-one faecal samples from 22 habituated gorillas and 74 samples from 15 habituated chimpanzees were analysed using Cytochrome-b PCR assay and coprological methods.

**Results:**

Overall, 26.4% of the analysed samples were positive for both *Plasmodium* spp. and strongylids. However, the results showed no significant impact of intensity of infections of strongylids on detection of *Plasmodium* DNA in gorilla and chimpanzee faeces.

**Conclusion:**

Bleeding caused by strongylid nematode *Necator* spp. cannot explain the presence of *Plasmodium* DNA in ape faeces.

## Background

Helminths are among the most ubiquitous parasite infections in primates and the distribution of helminth parasites is prominent in the areas where malarial infection occurs, resulting in high rates of co-infection [1]. A plethora of studies in humans have studied malaria and helminth co-infections with conflicting results [2]. In contrast, analogous studies in non-human primates (NHPs) have been restricted by the limitation of diagnosing malaria from blood and the obvious challenges associated with invasive sampling of wild primates. However, recent advances in molecular diagnostics enable malaria detection from non-invasively obtained faecal samples in primates [3, 4] and have paved the way for such non-invasive studies in the wild. Previously, studies in NHPs focused on the genetic diversity of *Plasmodium* spp. [3] and/or ecological aspects [5], no study has investigated co-infections with malaria and other pathogens.

Despite the pivotal role of non-invasive faecal sampling, little is yet known about the causal mechanism that lies behind the presence of ape *Plasmodium* DNA in faeces. Recently, a single experimental study demonstrated that the pre-erythrocytic stage of the malaria parasite can be detected in mouse faeces, and suggested that parasite DNA gets into the faeces via the bile duct [6]. However, it remains unclear if a similar mechanism applies to *Plasmodium* DNA entering ape faeces or if under natural conditions other mechanisms might be involved in the shedding of *Plasmodium* stages into the gastro-intestinal tract. Great apes are commonly infected by strongylid nematodes [7, 8] including hookworms [9]. Hookworms, *Ancylostoma duodenale* and *Necator* spp., can cause significant blood loss when the adult parasites attach themselves to the intestinal mucosa and while feeding [10], and thus their presence and consecutive bleeding in the intestine might be a plausible explanation for the detection of *Plasmodium* DNA in faeces.

## Methods

Since 2007 we have been systematically surveying parasite infections of humans, western lowland gorillas and other wildlife inhabiting Dzanga Sangha Protected Areas (DSPA), Central African Republic (CAR), as part of an ongoing health-monitoring programme [5, 9]. Previous research has shown that both gorillas in DSPA and chimpanzees in Kalinzu Forest Reserve (KFR), Uganda were infected with strongylid nematodes [9], (Hasegawa and Pafčo, unpublished data) and with malaria parasites *Plasmodium* spp. [3, 5]. In this study, co-infections with malaria and strongylid nematodes were explored in both gorillas and chimpanzees. The hypothesis was that a higher infection intensity of strongylid nematodes can result in higher detection rates of *Plasmodium* spp. in ape faeces.

In 2012 51 faecal samples from nine (n = 30 samples) and 13 (n = 21 samples) individuals were collected from two habituated gorilla groups (Makumba, Mayele) in DSPA, CAR. For further description of the field site and studied gorilla groups see Mapua et al. [5]. In 2014 74 faecal samples from 15 individuals from one habituated chimpanzee group (M group) were collected in KFR, Uganda. For a more detailed description of the study sites refer to Yasuoka et al. [11].

Most of the animals were sampled at least twice; however, six gorillas were sampled only once. The faecal samples were fixed immediately into RNA*later*™ (Ambion, Austin, TX, USA) for molecular detection of *Plasmodium* DNA and into 10% formalin for coprological analyses. Formalin-fixed samples were stored at ambient temperature, and RNA*later*-fixed samples were stored at 4 °C in a refrigerator at the base camp. The samples were subsequently shipped to the Czech Republic and those stored in RNA*later* were kept at −20 °C until DNA extraction. The methods for molecular detection of malaria, including DNA extraction, PCR, sequencing, and phylogenetic analyses are described in detail in Mapua et al. [5].

**Fig. 1:**
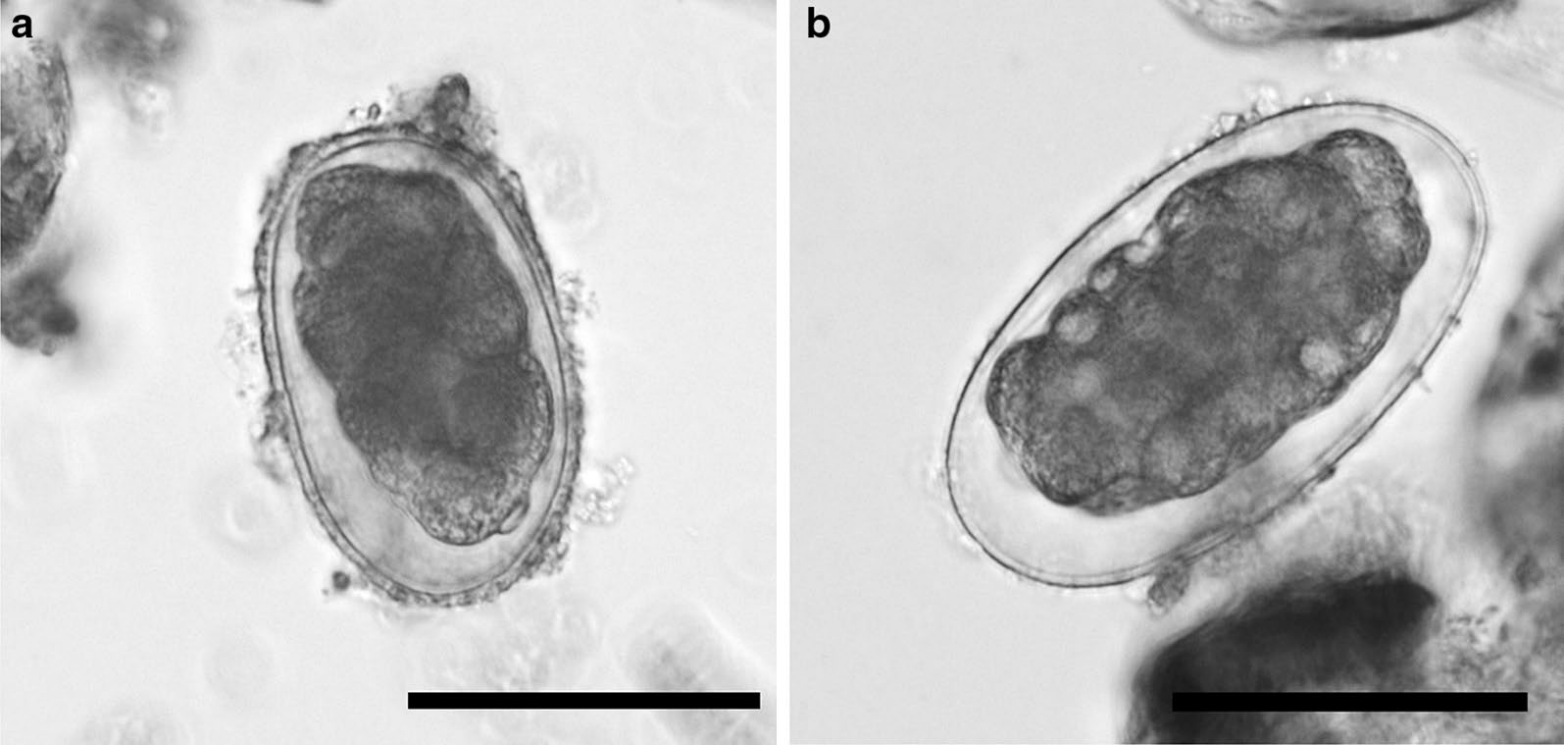
Strongylid eggs from feacal sample fixed in formalin. *Scale bar* 50 μm

For initial coprological examination Sheather’s flotation with modified sugar solution (s.g. 1.33) [12] and the sedimentation method [13] were used. To assess infection intensity of strongylid nematodes the number of eggs per gram of sediment (EPG) were quantified directly from the sediment [14]. The exact determination of strongylids to genus or even species level is mostly unreliable based on egg morphology alone. Previous morphological and molecular analyses of filariform larvae obtained from coprocultures from studied groups of both gorillas and chimpanzees showed the thin-walled strongylid eggs belonged to either the genus *Oesophagostomum* or *Necator* (Fig. 1: Hasegawa and Pafčo, unpublished data, [9]). Of the two, only *Necator* spp. are known to cause intestinal bleeding, however the thin-walled strongylid eggs were categorized as ‘strongylid nematodes/strongylids’ and counts may have included eggs of both *Oesophagostomum* and *Necator*. Morphological analyses of L3 larvae from pilot sets of coprocultures showed that all coprocultures contained *Necator* spp., however only 54 and 29% for gorillas and chimpanzees, respectively, contained larvae of *Oesophagostomum* spp. (Hasegawa and Pafčo, unpublished data). *Mammomonogamus* sp., the only strongylid eggs which can be easily distinguished by microscopy, can be also found in faecal samples of gorillas in DSPA [14]. *Mammomonogamus* spp. eggs were excluded from ‘strongylid nematodes/strongylids’ egg counts as adults of *Mammomonogamus* spp. occur in the respiratory tract of the host not in the gastro-intestinal [15].

Prevalence was defined as the number of positive samples for both *Plasmodium* DNA and strongylid eggs divided by the total of samples tested. Statistical analyses were performed using R software v 3.1.3; R Development Core Team 2015 [16, 17]. The effect of strongylid infection intensity on malaria detection in faeces was tested using generalized linear mixed effects models (GLMMs), with the presence of *Plasmodium* spp. as a binomial response variable (positive/negative for *Plasmodium* spp. DNA in faeces) and individual identity and host species (gorilla and chimpanzees) included as random factors. GLMMs were fitted by maximum likelihood with the binomial error distribution and implemented using the glmer function of the R package lme4.

## Results

**Fig. 2:**
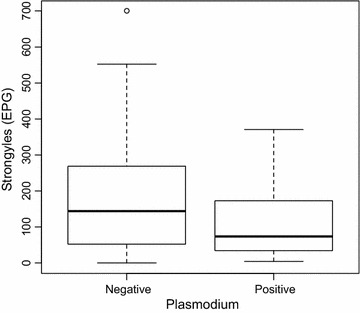
Box and whisker plot showing the association between the presence of malaria and distribution of strongylid eggs in the faeces

Results of the molecular analyses of *Plasmodium* spp. infecting western lowland gorillas in DSPA were reported in detail in Mapua et al. [5]. In eastern chimpanzees the host specific strains of *Plasmodium reichenowi*, *Plasmodium billbrayi* and *Plasmodium billcollinsi* were reported [18]. All examined chimpanzees and gorillas were positive for strongylid nematodes except three gorilla samples (see Appendix). Of the total 74 chimpanzees samples analysed, 12.16% (9 samples) were positive for both *Plasmodium* spp. and strongylid nematodes, in gorillas it was 47.06% (24 samples) from total of 51 samples. The mean intensity of strongylid infection in gorillas was 82.86 EPG (range 4–428) for malaria positive and 77.97 EPG (range 0–500) for malaria negative samples, in chimpanzees it was 263.13 EPG (range 51–370) for malaria positive and 281.90 EPG (range 13–1478) for malaria negative samples (Fig. 2). There was no significant effect of infection intensity of strongylid nematodes on detection rate in faeces for *Plasmodium* spp. DNA (z = −0.679, P = 0.497).

## Discussion

The results did not support the hypothesis that bleeding caused by strongylid nematode *Necator* spp. can explain the presence of *Plasmodium* DNA in western lowland gorilla and eastern chimpanzee faeces. The magnitude of *Necator* spp. pathogenesis and blood shedding is highly dependent on the number of adult worms, host immunity and concurrent infections with other nematodes [19]. To properly estimate infection intensity of a particular gastro-intestinal parasite species, invasive collection of adult parasites from the intestines of infected hosts is required, which is indeed impossible in wild endangered animals like great apes, with exception of necropsies. Due to individual and temporal variation in egg output, it is questionable whether the number of parasite eggs shed in faeces is linearly correlated with infection intensity [20]. However, several studies have found a linear relationship between faecal number of eggs and adult worm number in hosts [e.g., 20] and thus number of eggs has been used as a proxy for intensity of parasite infection also in non-invasive studies of non-human primates [21].

The results can be affected by the fact that it is impossible to distinguish eggs of *Necator* spp. from other strongylids, which are not associated with intestinal bleeding (as *Oesophagostomum* spp.) with the exception of *Mammomonogamus* sp. However, preliminary results indicate that *Necator* spp. are more prevalent in both studied ape populations and although *Oesophagostomum* infections are usually not associated with intestinal bleeding in humans, there is a report of rectal bleeding due to *Oesophagostomum brumpti* in a human [22]. However, in general, data about clinical manifestations and pathogenesis of any strongylid infections in wild non-human primates are scarce [23].

Taking an alternative perspective, *Plasmodium* pathogenesis itself may facilitate shedding of the parasites into the gastrointestinal tract [6]. It is known that the spleen plays a central role in phagocytosis of different pathogens including blood-stages of *Plasmodium* [24]. The liver is known as the site of pre-erythrocytic development of *Plasmodium* parasites and also participates in degradation and removal of the infected red blood cells from the circulation [24]. Recently, an experimental study on rodent malaria showed that *Plasmodium* DNA is detectable in the liver and gall bladder, as well as in faeces [6]. It was suggested that *Plasmodium* DNA derived from pre-erythrocytic stage parasites were released into the gastro-intestinal tract via the bile duct. Given that this experimental study was based on the inoculation of a high number of sporozoites and detection of *Plasmodium* DNA shortly after infection, it is questionable whether a similar phenomenon occurs in different hosts that are exposed to long-term infections with low parasitaemia levels under natural conditions. The inability to obtain wild ape blood samples, as well as predicting when the animals get infected, make difficult to suggest which *Plasmodium* stages could be detected in the ape faeces, and if *Plasmodium* DNA detected in this study underwent similar mechanism.

## Conclusions

Although no significant impact of strongylid infections on detection of *Plasmodium* DNA in faeces of wild apes was found, further studies are warranted to better understand the relationship between hookworms and malaria infections. Faecal and blood samples should be examined from sub-clinical malaria-positive humans with and without strongylid hookworms or the use an experimentally co-infected primate model to understand the possible role of hookworm infections in the detection of *Plasmodium* DNA in faeces.

## Appendix

See Table 1.
